# Calculation of the Date of Labour

**Published:** 1908-07-18

**Authors:** G. L. Brodhead


### CALCULATION OF THE DATE OF LABOUR.

SIR,—I am in receipt of the copy of the journal (June 20) in which you have been kind enough to refer to my article in a recent "Post Graduate." Your point is well taken, with reference to calendar or lunar months, but the fact is that I have always "thought" and spoken in terms of calendar months. It seems to me to be much simpler, and practically as good a method. Reference is so often made in my paper to eight and nine months, that one would naturally infer that calendar months were meant. The fundus is said to be so high at eight and nine months, but no mention is made anywhere of ten months. Again, in the table referring to dilatation of the os, there is no reference to a period beyond nine months, and while your criticism will be in future kept in mind, I believe readers would assume that calendar months were intended to be understood. Thanking you for the idea you have given me, and for the reference to my paper.

Yours very truly,
G. L. BRODHEAD.

